# Supplementary material for: Single cell level analysis of ATP release kinetics and cell fate following ultrasound targeted microbubble cavitation using microscopy techniques
Source: PLoS One. 2025 May 27;20(5):e0319318. doi: 10.1371/journal.pone.0319318 (PMC12111609; doi:10.1371/journal.pone.0319318)
Supplement: S1 Appendix — (DOCX) [file pone.0319318.s001.docx]

# S1 Appendix. Microfluidic device manufacturing

The microfluidic device consisted of two layers: the upper layer surface was molded, and the bottom layer was a simple flat surface. The mold of the upper layer was designed as described in the methods and machined in Poly(methyl-methacrylate) with a computer numerical control machine (Roland MDX-40A, Roland DGA Corporation, Irvine, CA, USA). The devices were manufactured in Polydimethylsiloxane (PDMS) (SYLGARD™ 184 Silicone Elastomer Kit, 4019862, DOW, Ellsworth Adhesives, ON, Canada). The silicone elastomer and the curing agent present in the kit were mixed homogeneously (1:10; w:w). 4.5 g were poured into the mold (molded part) and 40 g were poured into a Petri dish (diameter: 15 cm). The mold and the Petri filed with PDMS were sequentially placed in a vacuum chamber until complete PDMS degasification, and then in a stove at 80°C for 45 min (PR305220M Precision™ Compact Oven, Thermo Scientific™, Marietta, OH, USA) until curing. Both parts were de-molded delicately. The large PDMS piece in the Petri was cut into rectangular pieces of the same size as the molded parts with an additional margin of 5 mm on each side. The two PDMS layers were treated with an atmospheric plasma probe (Dyne-A-Mite 3D treater, Enercon Industries Corporation, Menomonee Falls, WI, USA) for 30 s and then gently pressed on one another for 10 s to ensure the adhesion of the two parts.
