# Supplementary material for: Single cell level analysis of ATP release kinetics and cell fate following ultrasound targeted microbubble cavitation using microscopy techniques
Source: PLoS One. 2025 May 27;20(5):e0319318. doi: 10.1371/journal.pone.0319318 (PMC12111609; doi:10.1371/journal.pone.0319318)
Supplement: S2 Appendix — (DOCX) [file pone.0319318.s002.docx]

# S2 Appendix. Acoustic alignment and transducer calibration

## Acoustic alignment

The acoustic alignment was performed with an in-house-designed 3D printed tunable device called waveguide (Fig A).


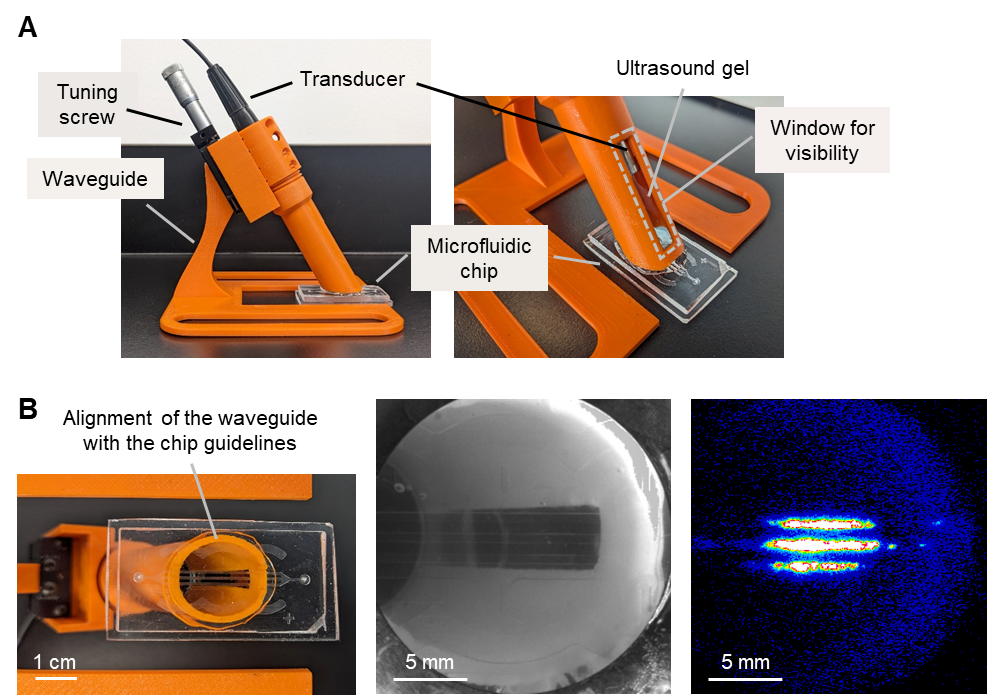


**Fig. Acoustic alignment.** (A) Waveguide description. (B) Left: Bottom view of the waveguide placed on the chip i.e. camera’s point of view. Middle: Image of the chip through the EMCCD camera with the room’s lights switched on. Right: Corresponding image through the EMCCD camera with the room’s lights switched off and bioluminescence signal after ultrasound treatment.

The waveguide was designed so that the transducer’s window was positioned at a 2.67 cm distance from the surface of the microfluidic device with an angle of 60° with the horizontal axis. The transducer was wedged inside the waveguide, and degassed ultrasonic gel was inserted with a long nozzle pipette. The waveguide was positioned with the help of the view from the EMCCD camera (Fig B – middle panel). The alignment across the three channels was done by placing the wave-guide contours following the transducer placement guidelines molded the microfluidic device (Fig B – left panel). The EMCCD camera had a large field of view (2 cm x 2 cm) encompassing most of the channel length (2.78 cm). Therefore, the alignment within the lengths of the channels was done by making sure the entire waveguide contour was in the camera field of view.

## Transducer calibration

The transducer was positioned at a distance of 2.67 cm and a right angle with a membrane hydrophone (HMB-0200 S/N: 1423, Onda Corporation, CA, USA) using an in-house 3-axis robotic arm. During calibration, the transducer and the hydrophone were immersed in a degassed water tank. The transducer was aligned with the hydrophone’s center and a PDMS piece of thickness 3.6 mm, representing the distance traveled by the US through the PDMS from the transducer to the cells, was positioned at an angle of 45° with the hydrophone surface. An attenuation of −0.43 ± 0.05 dB/MHz/mm (N = 12, 3 measures at 4 different pressures) was found for PDMS. The pressures indicated in this study are not corrected for the attenuation by PDMS.
