# Supplementary material for: Single cell level analysis of ATP release kinetics and cell fate following ultrasound targeted microbubble cavitation using microscopy techniques
Source: PLoS One. 2025 May 27;20(5):e0319318. doi: 10.1371/journal.pone.0319318 (PMC12111609; doi:10.1371/journal.pone.0319318)
Supplement: S3 Appendix — (DOCX) [file pone.0319318.s003.docx]

# S3 Appendix. Calibration factor for ATP quantification


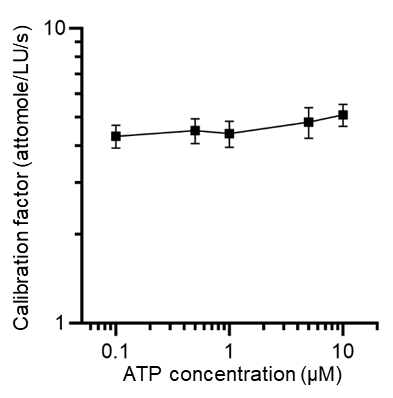


Calibration factor of the microfluidic chip as a function of the ATP concentration used to convert light unit measured into ATP concentration (Mean ± SD; N = 3).
