# Supplementary material for: Single cell level analysis of ATP release kinetics and cell fate following ultrasound targeted microbubble cavitation using microscopy techniques
Source: PLoS One. 2025 May 27;20(5):e0319318. doi: 10.1371/journal.pone.0319318 (PMC12111609; doi:10.1371/journal.pone.0319318)
Supplement: S4 Appendix — (DOCX) [file pone.0319318.s004.docx]

# S4 Appendix. Cell classification program description and validation

Fluorescence images were analyzed with an in-house MATLAB program divided into a segmentation followed by a classification step.

Cell segmentation on PI and calcein images was done with the watershed method applied to images where the background was removed with the adaptive Otsu method (1) available in MATLAB. Then, based on PI intensity and calcein/PI colocalization, the program classified each cell into three categories (dead, unaffected, sonoporated). Only PI-positive (PI+) cells were assumed to be dead. Only calcein-positive (calcein+) cells were assumed to be unaffected. For cells PI+ and calcein+ cells, a colocalization ratio was calculated as follows:

Let two images *CAL* and *PI* of the same size *M* × *N* with *V* gray levels in the range *[0; V-1]*. The gray value of the pixel with coordinates *(i, j)* is denoted by *CAL(i, j)* and *PI(i, j)*. The colocalization ratio (CR) is defined by:

$$\begin{aligned} \mathrm{CR} \left( \% \right)=\sum_{i=1}^{M} \sum_{j=1}^{N} \frac{\left( PI\left( i,j \right)\neq0 \right)\cap\left( CAL\left( i,j \right)\neq0 \right)}{\left( PI\left( i,j \right)\neq0 \right)}\times100 \end{aligned}$$

Cells with a CR higher than 90% (Fig A – class 1) or lower than 10% (Fig A – class 5), were respectively classified as sonoporated and dead regardless of their average nuclei PI intensity. Cells with a CR between 10% and 90% (Fig A – class 2 to 4) were classified considering both the PI intensity and the CR. Intermediate thresholds in PI intensity (*t_I_*) and CR (*t_CR%_*) were determined to classify these cells. These intermediate thresholds were calculated for every image with a two-weight clustering thresholding method described by Ashari *et al.* (WCT) (2). Cells where the CR was above *t_CR%_* and the PI intensity was under *t_I_* (Fig A – class 2) were classified as sonoporated and the others were classified as dead (Fig A – class 3 and 4).


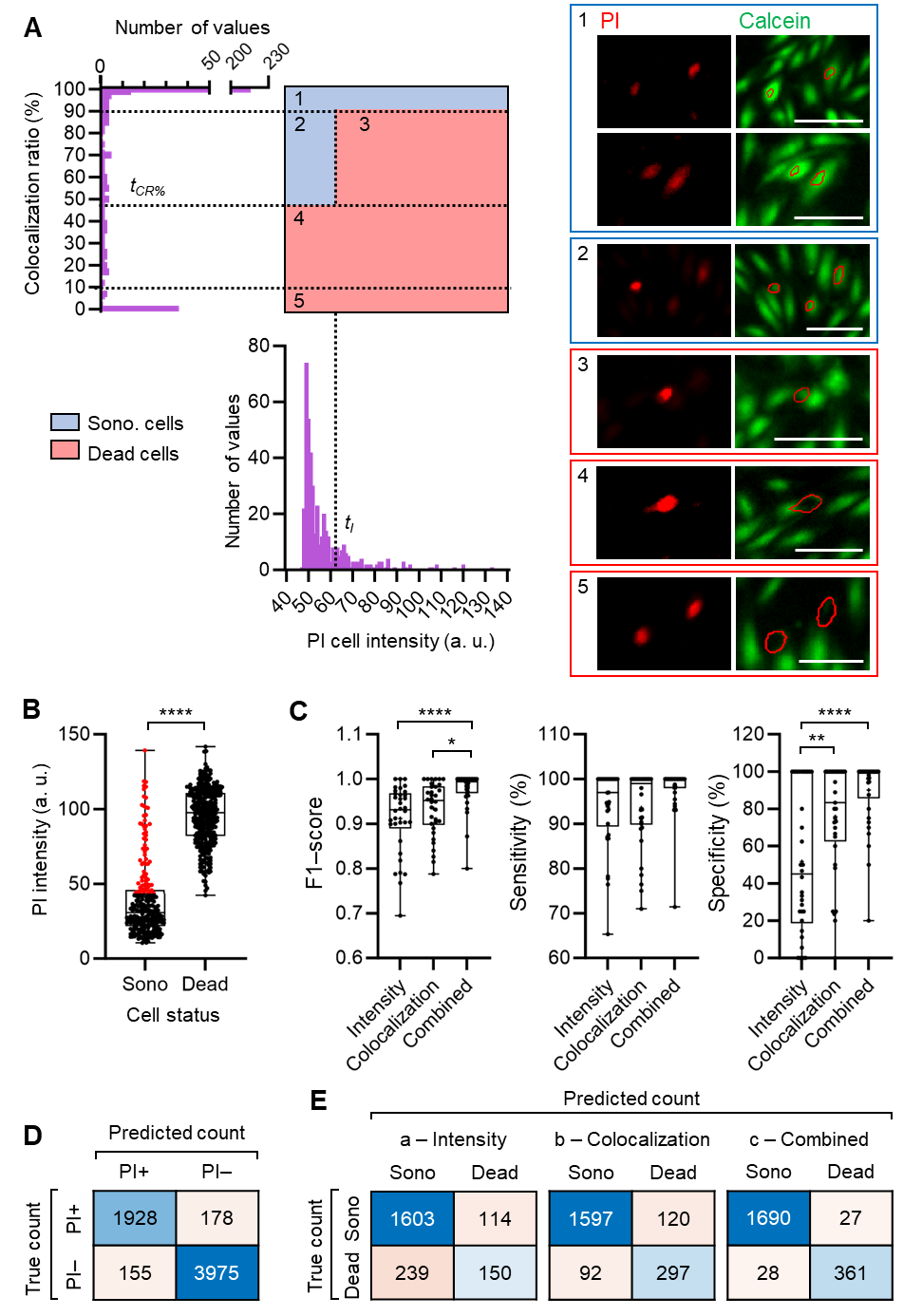


**Fig. Classification of cells on fluorescence image.** (A) Left. Classification table for PI-positive cells, based on the colocalization ratio (CR) between PI and calcein channels and the PI intensity. The two histograms of the PI intensity (at the bottom) and the CR (on the left) were plotted based on the results of a computation done on one image taken as an example where *t_I_* = 61 a. u. and *t_CR%_* = 48%. Right. Representative PI/calcein classification patterns corresponding to the 5 classes in the classification table. In the calcein image, the red contours were automatically segmented based on the PI images by our MATLAB program. Class 1: Sonoporation – Almost complete colocalization (CR above 90%) independently of the PI intensity. Class 2: Sonoporation – High colocalization (CR between *t_CR%_* and 90%) and a relatively low PI intensity ie. below *t_I_*. Class 3: Cell death – High colocalization (CR between *t_CR%_* and 90%) and a relatively high PI intensity, i.e. above *t_I_*. Class 4: Cell death – Relatively low colocalization (CR between *t_CR%_* and 10%) independently from the PI intensity. Class 5: Cell death – Almost no colocalization (CR below 10%) independently from the PI intensity. Scale bars 100 µm. (B) Average PI intensity in cell nuclei of dead (N = 328) and sonoporated (N = 240) cells. (C) F1-scores, sensitivity and specificity regarding the three different cell classifications. (D) Confusion matrix regarding the cell segmentation. (E) Confusion matrixes with a – the intensity-based classification, b – the colocalization-based classification, and c – the combined classification.

The program’s performance in cell segmentation and classification was assessed using the confusion matrix approach (3). This method compares predicted labels with actual labels, providing a comprehensive overview of accuracy and error types. The detected objects in the images were classified into four categories: true positives (TP), false positives (FP), true negatives (TN), and false negatives (FN). The program was executed on 34 cropped images (1000 µm × 1000 µm) randomly subsampled from the complete data set of fluorescence images obtained from the calcein/PI scans. On each image of the subsample data set, cells were counted and classified manually by the user. The program was also executed on the images with three different classification criteria: one computation with the criteria on the nuclei PI intensity alone (intensity-based classification), another with the criteria on the calcein/PI colocalization alone (colocalization-based classification), and the last one with both the criterions combined (combined classification). The performance of the segmentation was done on the combined classification computation with the two classes PI+ and PI– cells. A count of the non-detected PI+ cells (FN), and the over-segmented objects (FP) was done. The performance of the classification was done on the three different classifications of PI+ cells based on the two classes: sonoporated cells and dead cells. For every computation, the falsely dead (FN) and sonoporated (FP) cells were counted. The confusion matrices were obtained by summing the cell count from every image (Fig D & E). As there can be variabilities in every image (fluorophores intensity, cell density, PI+ cell density, etc.), the F1-scores, the sensitivity, and the specificity were calculated on every image regarding the segmentation steps and the three classifications (Fig C).

As demonstrated in the Figure S2B, PI intensity in cells’ nuclei (sonoporated N = 240; dead N = 328) was significantly higher in dead cells compared to sonoporated cells (95.5 ± 19.1 a. u. vs. 38.7 ± 25.3 a. u.; *p* < 0.0001; Student’s *t*-test) which was in favor of using the PI intensity as a criterion for cell classification.

All the F1-scores, the sensitivity and the specificity were the highest with the combined classification confirming the use of this classification method (Fig C). The summed confusion matrices also showed a refinement of the classification with the combined criterion (Fig E). One can note that the colocalization-based classification offered better scores than the intensity-based classification. This can be explained by the fact that most of the colocalization ratios were either 100% or 0% offering no other doubt to classify them as sonoporated and dead respectively (Fig A – colocalisation ratio histogram). Furthermore, the wide range of PI intensity in sonoporated cells occasionally overlapped with the PI intensity range observed in dead cells (Fig B – red dots).

Regarding the segmentation, the F1-score was on average 0.92 ± 0.06 which is generally considered excellent. This confirmed the methods used for the segmentation (Otsu for the background removal and watershed for the segmentation) were adapted for the processing of our images.

## References

1. Otsu N. A Threshold Selection Method from Gray-Level Histogram. IEEE TRANSACTIONS ON SYSTREMS, MAN, AND CYBERNETICS. 1979;62–3.

2. Ashari E, Armitage JC, Lessard RA, Hornsey RI, Lampropoulos GA. FPGA implementation of real-time adaptive image thresholding. In 2004 [cited 2023 Jul 11]. p. 410. Available from: http://proceedings.spiedigitallibrary.org/proceeding.aspx?doi=10.1117/12.566861

3. Pearson K. On the theory of contingency and its relation to association and normal correlation. In: Mathematical contributions to the theory of evolution. Dulau and Co. 1904. p. 1–34.
