# Supplementary material for: Single cell level analysis of ATP release kinetics and cell fate following ultrasound targeted microbubble cavitation using microscopy techniques
Source: PLoS One. 2025 May 27;20(5):e0319318. doi: 10.1371/journal.pone.0319318 (PMC12111609; doi:10.1371/journal.pone.0319318)
Supplement: S5 Appendix — (DOCX) [file pone.0319318.s005.docx]

# S5 Appendix. Estimation of total ATP in HUVEC cells using an independent method (Triton assay)

HUVEC cells were seeded in tissue culture-treated plates at different cell densities to reach a final density of 305 cells/mm^2^, 151 cells/mm^2^, 86 cells/mm^2^, and 43 cells/mm^2^. In all groups, cells were trypsinized from 3 wells and counted. In the other wells, the medium was removed and replaced by a solution of Triton X-100 (0.5% in double-distilled water) for 2 min at room temperature. The cell lysates were collected and diluted 100 times in double-distilled water to lower the ATP concentration within the detection range of the luminometer. The enzymatic activity of the LL enzyme was tested with ATP controls either diluted in double-distilled water alone or containing 0.005% Triton X-100 (ATP final concentration: 100 nM). The volume of these cells was also estimated as a prism of height 5 μm to assess the intracellular ATP concentration.

The cell surface of 100 cells was measured in each of four fluorescence images, in which the cell density was 301 cells/mm^2^, 148 cells/mm^2^, 87 cells/mm^2^, and 55 cells/mm^2^ i.e. at the nearest density of 305 cells/mm^2^, 151 cells/mm^2^, 86 cells/mm^2^, and 43 cells/mm^2^ respectively.


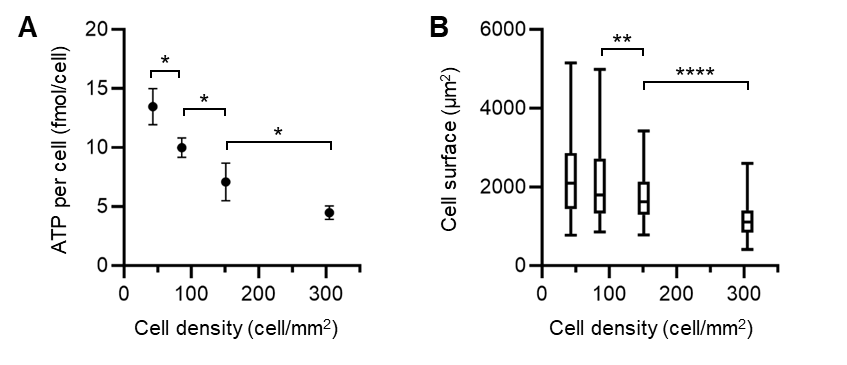


**Fig. Intracellular ATP.** (A) Estimation of the ATP quantity in HUVEC cells (Mean ± SD). (B) Estimation of the HUVEC cell surfaces.

The estimated intracellular ATP decreased from 13.5 ± 1.5 fmol/cell to 4.5 ± 0.5 fmol/cell with increased cell density (Fig A). Additionally, the average cell surface decreased and appeared less spread as the cell density increased (Fig B). The amount of ATP in HUVEC cells has previously been estimated in the fmol range (1), which is in the lower range of our findings. The estimation of the intracellular ATP concentration yielded on average 1.13 ± 0.68 mM, 0.85 ± 0.61 mM, 0.77 ± 0.52 mM, 0.78 ± 0.39 mM for cell densities of 43 cells/mm^2^, 86 cells/mm^2^, 151 cells/mm^2^, and 305 cells/mm^2^ respectively. These values are in the lower concentration range found in the literature, which varies between cell types but is typically in the mM range (2–4).

## Reference

1. Wang X, Ackermann M, Neufurth M, Wang S, Li Q, Feng Q, et al. Restoration of Impaired Metabolic Energy Balance (ATP Pool) and Tube Formation Potential of Endothelial Cells under “high glucose”, Diabetic Conditions by the Bioinorganic Polymer Polyphosphate. Polymers. 2017 Nov 4;9(11):575.

2. Bonora M, Patergnani S, Rimessi A, De Marchi E, Suski JM, Bononi A, et al. ATP synthesis and storage. Purinergic Signal. 2012 Sep;8(3):343–57.

3. Greiner JV, Glonek T. Intracellular ATP Concentration and Implication for Cellular Evolution. Biology. 2021 Nov 12;10(11):1166.

4. Schwiebert EM, Zsembery A. Extracellular ATP as a signaling molecule for epithelial cells. Biochim Biophys Acta BBA - Biomembr. 2003 Sep;1615(1–2):7–32.
